# First report and molecular characterization of the dagger nematode, *Xiphinema oxycaudatum* (Nematoda, Dorylaimidae) from South Africa

**DOI:** 10.3897/zookeys.894.35281

**Published:** 2019-12-03

**Authors:** Fisayo Y. Daramola, Rinus Knoetze, Antoinette Swart, Antoinette P. Malan

**Affiliations:** 1 Department of Conservation Ecology and Entomology, Private Bag X1, Matieland 7602, Stellenbosch, South Africa Department of Conservation Ecology and Entomology Stellenbosch South Africa; 2 Plant Protection Division, Agriculture Research Council (ARC) Infruitec-Nietvoorbij, Private Bag X5026, Stellenbosch 7599, South Africa Plant Protection Division, Agriculture Research Council Stellenbosch South Africa; 3 Nematology Unit, Biosystematics Division, ARC-Plant Protection Research Institute, Private Bag X134, Queenswood 0121, South Africa ARC-Plant Protection Research Institute Queenswood South Africa; 4 Department of Zoology, University of Johannesburg, PO Box 524, Auckland Park, Johannesburg 2006, South Africa University of Johannesburg Johannesburg South Africa

**Keywords:** *coxI*, D2D3, honeybush, molecular identification

## Abstract

Plant-parasitic nematodes of the genus *Xiphinema* Cobb, 1913 comprise a complex group of nematode species, some of which are important vectors of plant viruses. During a field survey to determine the soil health of an abandoned honeybush (*Cyclopia genistoides*) monoculture, a high density of the dagger nematode, *Xiphinema oxycaudatum* Lamberti & Bleve-Zacheo, 1979 (Nematoda, Dorylaimidae), was observed in soil around the roots of honeybush plants in an abandoned farmland at Bereaville, an old mission station in the Western Cape province of South Africa. Soil samples were taken from the rhizosphere of plants and nematodes were extracted from the soil using a modified extraction tray method. Specimen of the dagger nematodes were processed for scanning electron microscopy, morphological and molecular analysis. Molecular profiling of the nematode species was done in order to give an accurate diagnosis and to effectively discriminate the nematode from other species within the *Xiphinema americanum* group. Phylogenetic analysis based on the D2D3 expansion segment of the 28S gene supported a close relationship of species within the *americanum* group, however, the protein-coding cytochrome oxidase (*coxI*) of the mitochondrial gene provided a useful tool for distinguishing the nematode from other species within the group. This study represents the first report of *X. oxycaudatum* from South Africa.

## Introduction

Dagger nematodes, belonging to the *Xiphinema americanum*-group, are economically important nematodes that may cause damage to agricultural crops, by means of direct feeding on plant roots and in transmitting plant viruses. *Xiphinema oxycaudatum* Lamberti & Bleve-Zacheo, 1979 (Nematoda, Dorylaimidae) is a polyphagous and cosmopolitan nematode, which was first described from the rhizosphere of oil palm, *Elaies guineensis* in Nigeria (Lamberti and Bleve-Zacheo 1979). A high population of this nematode species were found in soil around honeybush (*Cyclopia genistoides*), in an abandoned farmland (-34.0516, 19.5174) at Bereaville in the Western Cape province, South Africa.

Although many nematode species in the *X. americanum*-group are widespread in distribution, *X. oxycaudatum* is localized in Africa with a few reports from Asia and South America (Lamberti et al. 2000; Fadaei et al. 2003; Oliveira et al. 2003; Chen et al. 2005). In South Africa, *Xiphinema* species have been listed as one of the most common and abundant plant parasitic nematodes causing damage on grapevines and woody plants (Fourie et al. 2017). However, only a few species belonging to the *Xiphinema americanum*-group have been reported in the country, some of these include; *X. americanum*, *X. brevicolle*, *X. diffusum*, *X. incognitum*, and *X. pachtaicum* (Lamberti et al. 1995, 2002).

Honeybush is an exclusive African herbal tea with a distinctive honey aroma and it is a rich source of compounds with antimutagenic properties (Kokotkiewicz and Luczkiewics 2009). There is an increasing demand for honeybush production in South Africa, due to increased awareness of the health benefits obtainable from this unique tea (SAHTA 2011).

In this study, the dagger nematodes found in soil around honeybush were identified with a combination of traditional morphological characterization and molecular techniques, based on the D2D3 expansion segment of the 28S gene and the protein-coding cytochrome oxidase (*coxI*) of the mitochondrial gene.

## Methods

### Sampling, nematode isolation, and processing

Soil samples were collected from three plots on the honeybush farmland, with five composite samples taken from each plot. Samples were taken from the rhizosphere of the plants, a depth of about 8 cm into the soil. Nematodes were extracted from the soil using a modified Whitehead and Hemming (1965) tray method and examined under a high-power compound microscope. Nematode specimens from a previously identified population of *Xiphinema americanum* Cobb, 1913 from a grapevine farm in the Western Cape was also included in the study. Nematodes were counted using a stereomicroscope and specimens collected for morphological study, scanning electron microscopy (SEM), and for molecular characterization of nematode species.

### Light and scanning electron microscope observations

For light microscopy, nematode specimens were mounted on glass slides and observed under a compound microscope. Morphological characters were measured and light micrographs were taken with a Zeiss Axioskop 40 compound microscope equipped with a drawing tube. Adult females and juveniles were observed. Some of the morphometric features that were measured include total body length, oesophageal length, body diameter, stylet lengths (odontostyle and odontophore), lip region diameter, distance of basal guide ring from anterior, distance from anterior end to the vulva, width at vulva, and the tail length (Table 2). These measurements were used to calculate the characters; a, b, c, c’ and V.

**Table 1.** Primer combination.

**Primer code**	**Direction**	**Sequence (5'–3')**	**Amplified gene**	**References**
D2A	Forward	ACA AGT ACC GTG AGG GAA AGT TG	28S rRNA	Nunn 1992
D3B	Reverse	TCG GAA GGA ACC AGC TAC TA		Nunn 1992
ITS1	Forward	TTGATTACGTCCCTGCCCTTT	ITS rRNA	Vrain et al. 1992
P28S	Reverse	TTTCACTCGCCGTTACTAAGG-		Vrain et al. 1992
CO1F	Forward	GATTTTTTGGKCATCCWGARG	COI	He et al. 2005
CO1R	Reverse	CWACATAATAAGTATCATG	COI	
XIPHR1	Reverse	ACAATTCCAGTTAATCCTCCTACC	COI	Lazarova et al. 2006
XIPHR2	Reverse	GTACATAATGAAAATGTGCCAC	COI	Lazarova et al. 2006

**Table 2.** Morphometrical data of *Xiphinema oxycaudatum* from South Africa. Measurements are in µm, except where stated otherwise, in the form of: mean ± standard deviation (range).

	**Female**	**Pre-adult**	**Stage before pre-adult**
n	11	6	1
L (mm)	1.80 ± 10.52 (1.60–1.94)	1.42 ± 8.79 (1.33–1.52)	128.5
a	46.87 ± 4.37 (39.9–55.3)	37.46 ± 2.90 (33.6–40.9)	42.8
b	6.14 ± 0.56 (4.8–6.9)	4.93 ± 0.78 (3.5–5.7)	5.2
c	50.34 ± 2.96 (45.4–55.3)	37.28 ± 4.48 (29.9–42.2)	37.3
c’	1.43 ± 0.08 (1.3–1.6)	1.58 ± 0.10 (1.4–1.7)	1.6
V	49.82 ± 1.44 (47.8–52.4)	–	–
Odontostyle length	78.41 ± 5.12 (71–84)	64.9 ± 2.84 (61–68)	55
Odontophore length	56.14 ± 5.4 (46–66)	47.5 ± 0.84 (47–49)	43
Total stylet length	135.55 ± 5.41 (129.5–149.5)	112 ± 3.33 (111–117)	98
Replacement odontostyle length	–	78.78 ± 3.87 (74–85)	59.5
Anterior to guide ring	67.36 ± 2.84 (64–73)	55.5 ± 2.89 (50.5–58–73)	51.5
Tail length	35.82 ± 2.74 (31–41)	37.00 ± 4.70 (31–44.5)	34.5
h (hyaline portion of tail); also J	12.91 ± 1.61 (10.5–15.5)	9.92 ± 1.02 (9–11)	11
h % (hyaline portion/tail length)	36.08 ± 3.85 (29–40.3)	26.93 ± 1.95 (23.7–29)	31.9
Lip region diameter	12.86 ± 0.87 (11.5–13.5)	11.5 ± 0.54 (11–12)	11
Lip region height	5.86 ± 0.32 (5.5–6.5)	5.42 ± 0.38 (5–6)	4.5
Body diameter at guide ring	28.64 ± 1.80 (26–31.5)	25.75 ± 2.95 (23.5–31.5)	24
Body diameter at base of pharynx	36.20 ± 2.52 (33–42)	34.67 ± 4.03 (33–41)	29
Body diameter at vulva or mid-body for juvenile	39.18 ± 2.57 (36.5–44)	37.90 ± 5.19 (31–44)	30
Body diameter at anus	25.18 ± 1.97 (20.5–27.5)	23.42 ± 2.25 (20–26)	21
Body diameter at beginning of hyaline portion of tail	13.50 ± 1.22 (11.5–16)	10.67 ± 0.61 (10–11.5)	9
Pre-rectum length	103.85 ± 47.37 (47–214)	55; 70	–
Rectum length	20.14 ± 4.61 (15–31.5)	23.13 ± 7.49 (17–34)	–
Vagina length	14.68 ± 1.01 (12.5–16)	–	–

Specimen samples for SEM were handpicked, fixed overnight in 2% Glutaraldehyde and dehydrated in increasing concentrations of ethanol. The nematode specimens were chemically dried with Hexamethyldisilizane (HMDS) in a fume hood and kept in a desiccator overnight. Nematodes were mounted on double-sided carbon tapes on Al stubs and were sputter coated with Pd/Au at a thickness of 100Ǻ layer for 10 min.

A Zeiss Merlin FESEM (Carl Zeiss Microscopy, USA) was used to generate electron images at 3kV accelerating voltage using InLens SE and SE2 detection and a probe current of 100–150 pA. Images were captured in TIF format using a pixel averaging noise reduction algorithm.

### DNA extraction, PCR, and sequencing

DNA was extracted from single adult female nematodes using a modified method of Nguyen (2007). The polymerase chain reaction (PCR) to confirm the identity of the nematode specie was carried out by the amplification of the internal transcribed spacer (ITS) region, the D2D3 expansion segment of the 28S gene of the ribosomal DNA, and the portion of the cytochrome oxidase (*coxI*) gene of the mitochondrial DNA. PCR of the ITS region was carried out as described by Chen et al. 2005 using KAPA2G 40 Robust HotStart ReadyMix (KAPA Biosystems) with the primer combination of S-ITS1 (5'-TTGATTACGTCCCTGCCCTTT-3') and 28S (5'-TTTCACTCGCCGTTACTAAGG-3'). Amplification was carried out in a thermal cycler with the following cycling condition; 1 cycle at 94 °C for 4 min, followed by 30 cycles at 94 °C for 30 sec, 52 °C for 30 sec, and 72 °C for 2 min 30 sec, and ending with one cycle at 72 °C for 7 min and ﬁnally kept at 4 °C. PCR amplification of the D2-D3 expansion segments of the 28S rDNA gene was carried out with the primer set D2A (5'-ACA AGT ACC GTG AGG GAA AGT TG-3') and D3B (5'-TCG GAA GGA ACC AGC TAC TA-3') with the cycling condition of 4 min at 94 °C, followed by 35 cycles of 1 min at 94 °C, 1 min at 55 °C, and 1 min 30 sec at 72 °C, and a ﬁnal extension at 72 °C for 10 min (Orlando et al. 2016). The portion of the partial *coxI* of the mitochondrial gene was amplified using a primer combination of the forward primer, COIF (5'-GATTTTTTGGKCATCCWGARG-3') with the reverse primer, XIPHR2 (5'-GTACATAATGAAAATGTGCCAC-3') as described by Lazarova et al. (2006). The thermal condition includes 1 cycle of 94°C for 1 min, 50 °C for a further 1 min and 72 °C for 2 min. This was followed by 40 cycles of 94 °C for 1 min, 45 °C for 1 min and 72 °C for 2 min. PCR was ended with a ﬁnal extension phase of 94 °C for 1 min, 45 °C for 1 min and 72 °C for 5 min.

### Sequence and phylogenetic analysis

PCR products were purified using the Nucleo-Fast Purification System (Macherey Nagel, Waltham, Massachusetts, USA). Sequencing of the purified DNA was performed in both directions with the Big Dye Terminator V1.3 sequencing kit, followed by the use of electrophoresis on the 3730× 1DNA Analyser (Applied Biosystems) at the DNA Sequencing Unit (Central Analytical Facilities, Stellenbosch University). The Software CLC Main Workbench 7.3 (http://www.clcbio.com) was used for sequence assembly and editing. Newly obtained partial *coxI* sequences of *X. oxycaudatum* and *X. americanum* were deposited on the GenBank database with accession numbers MK211480 and MK956813 respectively. DNA sequences obtained for the D2D3 expansion segment of *X. oxycaudatum* was also deposited with accession numbers MK947997, MK966417, and MK988554.

The newly obtained DNA sequences were used for BLASTN (Altschul et al. 1997) comparison against GenBank sequences. DNA sequences from the top BLASTN matches, and other nematode sequences, were downloaded from GenBank and aligned using Multiple Alignment using Fast Fourier Transform (MAFTT).

The evolutionary history of the *coxI* region of the mitochondrial gene and D2D3 expansion segment of the 28S gene was inferred using the maximum parsimony (MP). The most parsimonious tree is shown. Evolutionary analyses were conducted in MEGA X version 10.0.5 (Kumar et al. 2018) and the confidence intervals for the various branching patterns in the trees were measured using bootstraps (Felsenstein 1985) with 1000 replicates. Estimates of the evolutionary divergence between sequences was done using pairwise distance analysis.

## Results

*Xiphinema oxycaudatum* was observed in high numbers from samples taken from the abandoned honeybush farmland with a mean population density of about 510/250 cm^3^ soil.

**Figure 1.**
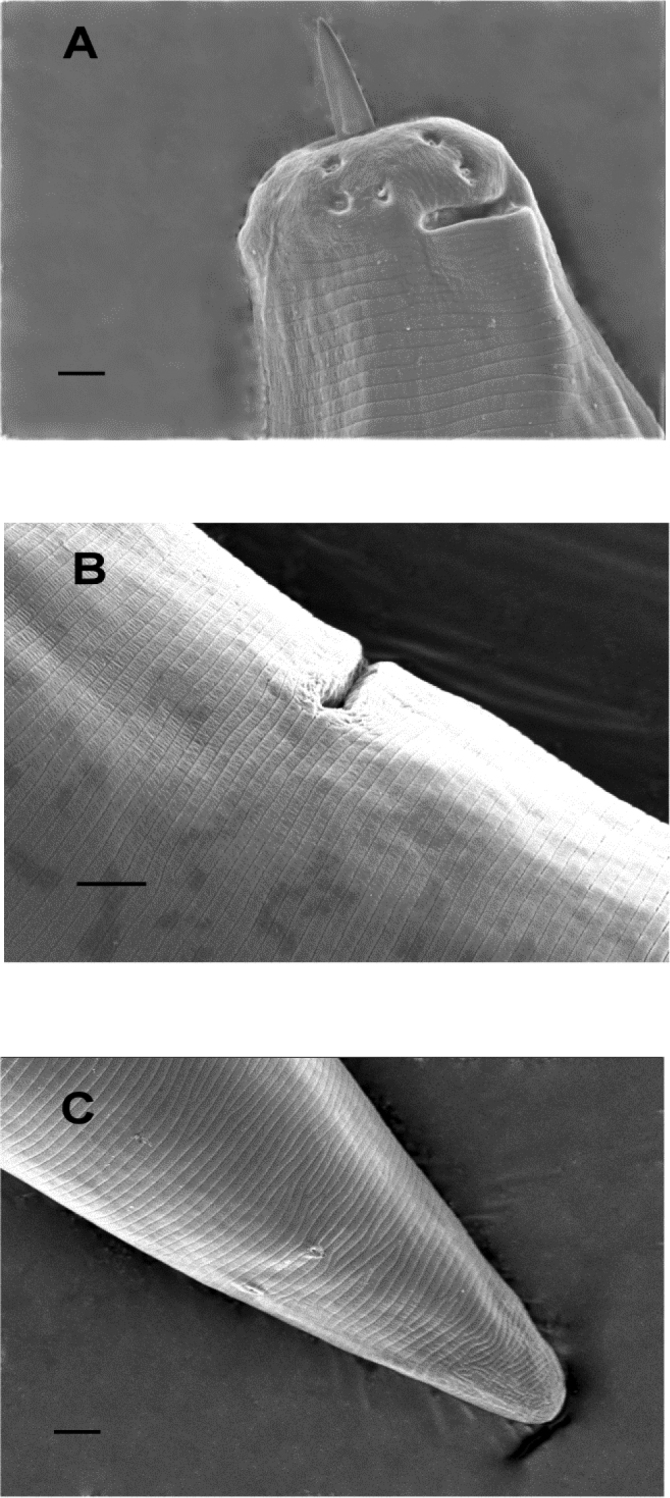
Scanning electron micrographs of *Xiphinema oxycaudatum***A–C** head region with stirrup-shaped amphidial pouch and slit-like aperture, vulva opening and tail showing a caudal pore. Scale bars: 2 µm.

Observations with SEM provided detailed information on some intrinsic features of the nematode such as the stirrup-shaped amphidial pouch, slit-like aperture, caudal pores and vagina opening (Fig. 1).

The morphological features of the nematodes are similar to those described from Nigeria (Lamberti and Bleve-Zacheo 1979; Bos and Loof 1983). Both adult females and juveniles were observed. The habitus of the nematodes are spiral or C-shaped with a head that is slightly offset. Adult females are between 1600–1800 µm long. They are more ventrally curved at the posterior end than the anterior. The vulva is located slightly above 50% of the body length; the ovary is amphi-didelphic with long oviduct and short uteri. The tail is conoid with bluntly rounded terminus. The juvenile stages are similar to adult females, but with a smaller body size. They also possess more pointed and sharper conoid tails. No male was found.

### Description

**Figure 2.**
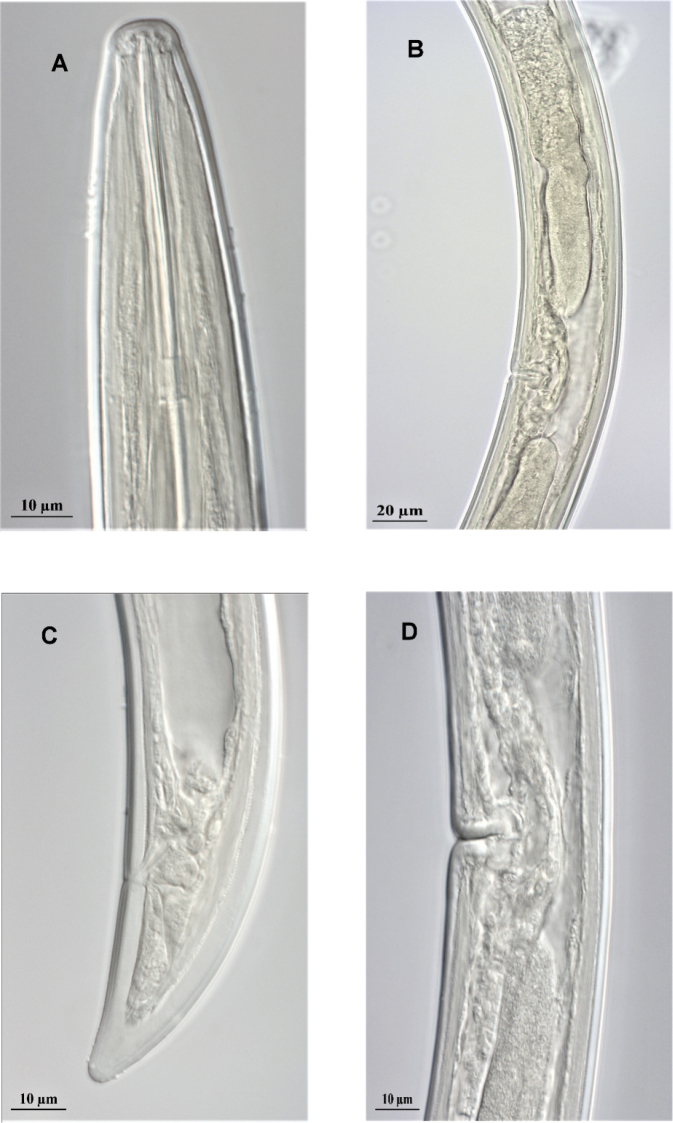
Light microscopy of *Xiphinema oxycaudatum***A–D** head region, female reproductive system with didelphic ovary, tail region and vulva. Scale bars: 10 µm (**A, C, D**), 20 µm (**B**).

Female: Body strongly curved ventrally into close C-shape. Cuticle 2.7 µm wide at mid-body, 6.5 µm at dorsal side of tail, radial striations visible on tail end. Lip region demarcated from body by slight depression (Fig. 2). Position of pharyngeal gland nuclei and outlets (as percentage of bulb length): DO = 9.21 (6.5–11); DN = 15.05 (10.1–18.2); DN–DO = 5.83 (3.6–7.3); SN1 = 59.07 (49.3–67.8); SO = 64.54 (49.3–74.3); SN1–SO = 5.42 (0–11); SN2 = 62.86 (59.1–69.3); SN2–SO = 11.25 (5–18.3). Neck region 288.75 ± 20.16 (265–310) µm long; cardia small, hemispherical to conoid in shape. Female reproductive system typical of *X. americanum* lineage (ovaries with symbionts, long oviducts, short uteri), each branch about two corresponding vulva diameters long. Tail conoid, dorsally convex, ventrally slightly arcuate with rounded terminus, two caudal pores on each lateral side.

### Relationship

The specimens from South Africa agree well with the type description of *X. oxycaudatum* (Table 2) but are slightly longer (1.6–1.94 mm vs 1.5–1.7 mm); the vulva is situated more anterior in one specimen (47.8% vs 51–54%) and have a wider head region (11.5–13.5 µm vs 9–10 µm). However, the South African specimens are closer to the description of *X. oxycaudatum* from Iran (Fadaei et al. 2003) especially in the body length (1.6–1.9 mm in Iranian specimens) and more anterior position of vulva in some females (45.5–54% in Iranian specimens). The wider head region in the South African specimens are considered to be an intraspecific variation. The pre-adult stage juvenile from South Africa agrees well with the description of this stage described from Iran (Fadaei et al. 2003). One juvenile was found, which apparently falls in a stage before the pre-adult juvenile. It can be distinguished from the pre-adult stage, by the shorter replacement odontostyle (59 µm vs 74–84 µm in pre-adult juvenile). The specimens from South Africa are also near *X. peruvianum* Lamberti & Bleve-Zacheo, 1979, but can be distinguished by the shorter odontostyle (71–84 µm vs 85–92 µm and the shape of the tail (gradually tapered, conoid vs not so gradually tapered, almost subdigitate).

**Figure 3.**
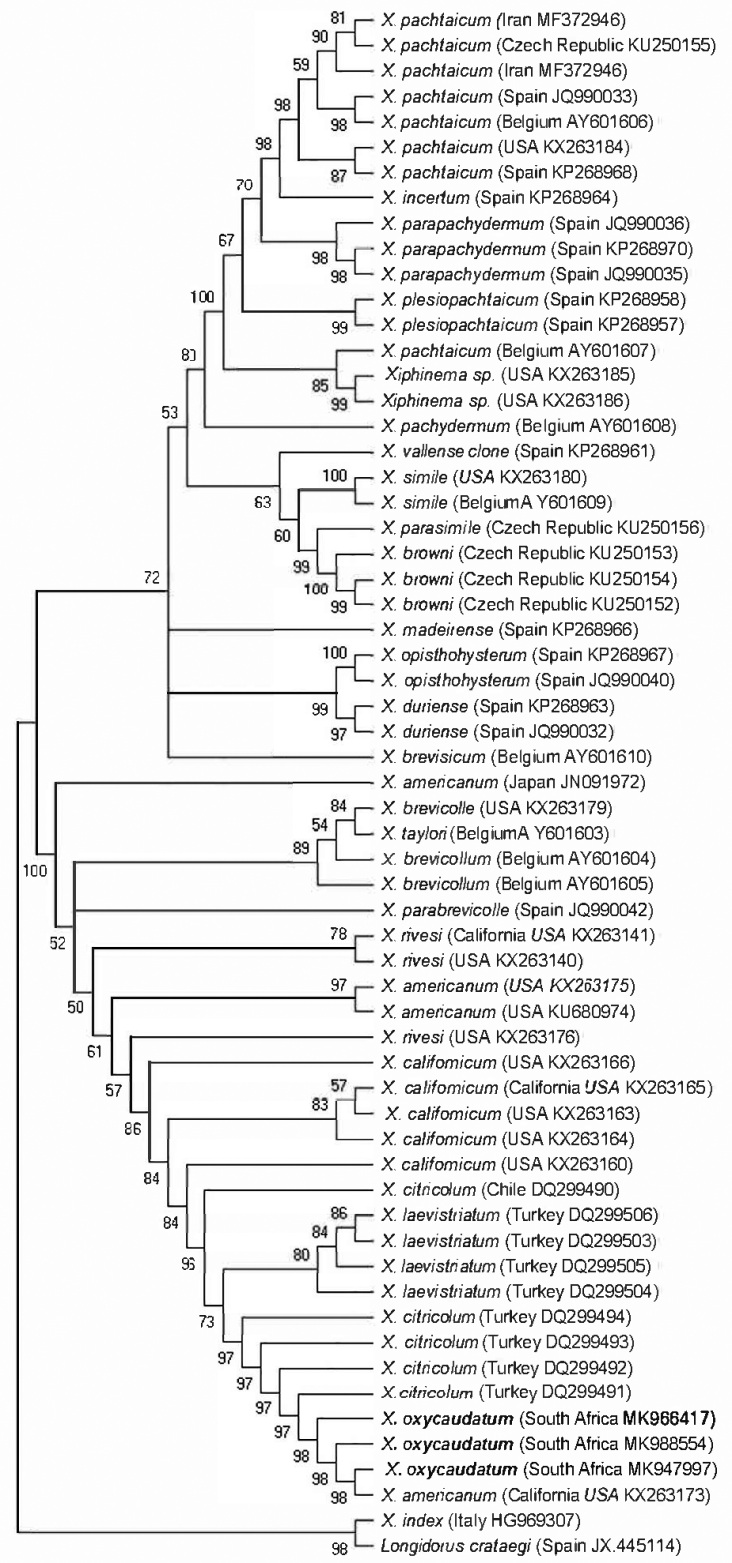
Phylogenetic relationship within species of the *Xiphinema americanum*-group, based on analysis of the D2D3 regions with maximum parsimony (MP) using *Xiphinema index* and *Longidorus crataegi* as outgroups. Newly obtained sequence is indicated by bold letters.

**Figure 4.**
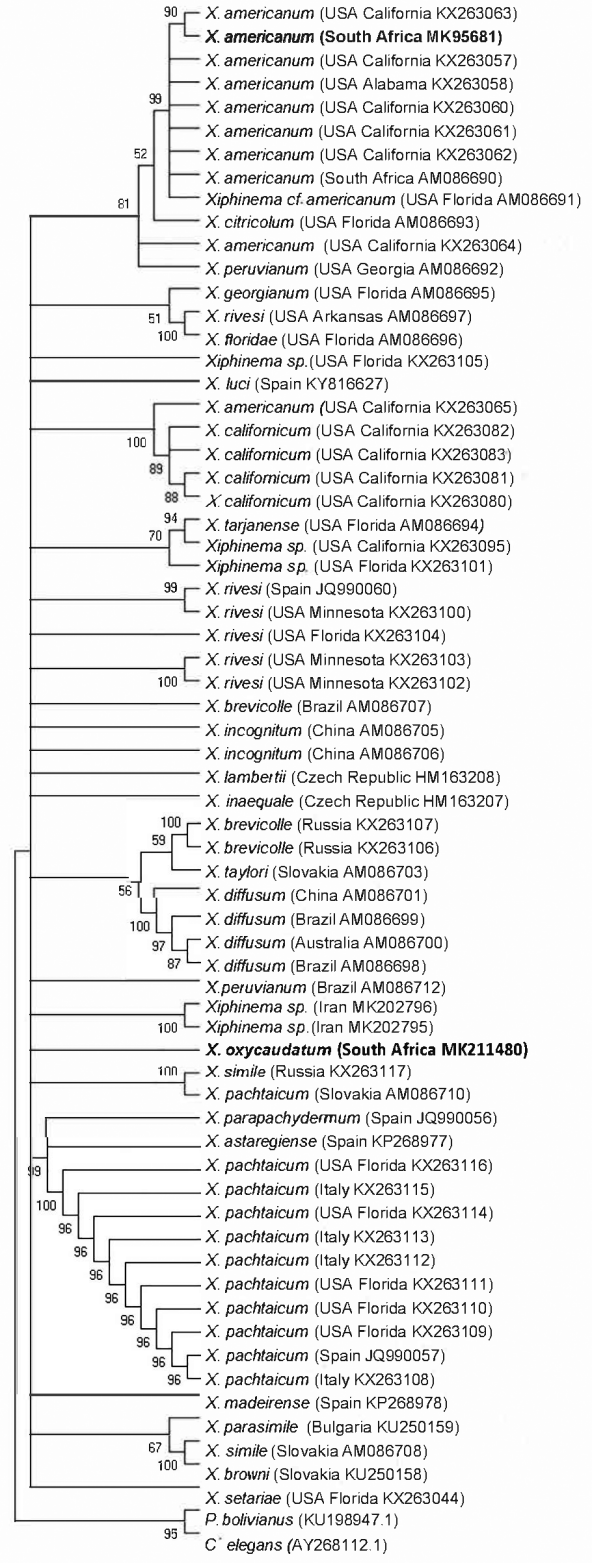
Phylogenetic relationship within species of the *Xiphinema americanum*-group, based on analysis of the *coxI* regions with maximum parsimony (MP), using *Pratylenchus bolivianus* and *Caenorhabditis elegans* as outgroups. Newly obtained sequences are indicated by bold letters.

The phylogenetic relationships within the *X. americanum*-group species inferred from the analysis of D2D3 expansion segments of 28S and the partial mitochondrial *coxI* gene using MP are given in Figures 3 and 4 respectively. The D2D3 alignment was 710 base pairs long and included 59 *X. americanum*-group sequences with two outgroup sequences (*X. index* and *Longidorus crataegi*). Phylogenetic analysis of the D2D3 expansion region revealed a high similarity of almost 100% with some species in the *americanum*-group. Nearly identical sequences were obtained from the studied species, with interspeciﬁc divergence ranging from 0 to 0.25%. The MP tree showed two supported clades. Clade I (72%) includes: *X. pachtaicum*, *X. incertum*, *X. pachydermum*, *X. parapachydermum*, *Xiphinema* sp., *X. simile*, *X. browni*, and other nematode species. Clade II (100%) comprised of *X. brevicolle* species complex (Orlando et al. 2016): *X. citricolum*, *X. americanum*, *X. californicum*, *X. rivesi*, *X. laevistriatum*, and other species. Relationship within this clade was not well resolved. Intra-specific variation with about 2–3 indel events was also observed in the *X. oxycaudatum* sequences. The genetic relationship of the newly obtained sequences with reference sequences obtained from the National Centre for Biotechnology Information (NCBI) is illustrated in Figure 3.

Species delimitation of *X. oxycaudatum* within the *X. americanum* group was achieved by analysing the *coxI* sequence alignment which comprised of 66 *X. americanum* group sequences and two other sequences, *Pratylenchus bolivianus* and *Caenorhabditis elegans* as outgroups. The alignment length was 298 base pairs long. Although there was no available sequence of the partial *coxI* gene of *X. oxycaudatum* on the NCBI database for comparison, the sequence showed a similarity of 86.19% and 82.48% with *X. peruvianum* and *X. rivesi* respectively. The pair-wise distance of *X. oxycaudatum* to the closely related Brazilian population of *X. peruvianum* is 245 base pairs differences (Table 3). Newly obtained *X. americanum* sequence showed a high similarity of 98.84% to the South African isolate (AM086690) with only four nucleotide differences. Estimates of the evolutionary divergence between the newly obtained sequence and some closely related ones is shown in Table 3. The number of base differences per sequence from between sequences are indicated.

**Table 3.** Pairwise distances of *COI* regions between *Xiphinema oxycaudatum* and some closely related sequences within the *Xiphinema americanum* group. The number of base differences per sequence from between sequences are shown.

	Species	1	2	3	4	5	6	7	8	9	10	11	12	13	14	15	16	17	18	19	20	21	22	23	24	25	26	27	28	29	30	31
1	X._ oxyca udatum_(South_Africa_M K211480)																															
2	X._rivesi_(USA_Florida_KX263104)	98																														
3	Xiphinema _sp._(USA_Florida _KX263101)	99	52																													
4	X._ tarjanense_ (USA_Florida_AM086694)	103	45	32																												
5	Xiphinema _sp._(lran_MK 202796)	105	56	66	56																											
6	X._georgianum_(USA_Florida_AM086695)	106	56	65	56	65																										
7	X._incognitum_(China_AM086705)	107	68	63	58	65	72																									
8	X._brevicolle_(Russia_KX263107)	107	76	68	62	62	72	55																								
9	X._rivesi_(Spain_JQ990060)	110	53	47	53	57	75	61	70																							
10	X._lambertii_ (Czech_Republic_H M163208)	112	77	74	79	72	75	63	62	73																						
11	X._brevicolle_(Brazil_AM086707)	118	69	70	74	69	81	66	77	77	82																					
12	X._luci_(Spa in_KY816627)	120	65	70	63	67	63	78	81	61	74	82																				
13	X._taylori_(Slovakia_AM086703)	120	69	66	70	61	69	71	43	72	71	81	71																			
14	X._ citricolum_(USA_Florida _AM086693)	122	61	58	59	57	67	68	67	68	71	79	59	73																		
15	X._florida e_ (USA_Florida_AM086696)	127	69	72	64	72	64	82	81	78	87	85	64	81	63																	
16	X._rivesi_ (USA_Arkansa s_AM086697)	128	69	70	65	70	66	84	79	74	85	85	62	81	69	6																
17	X._diffusum_(China_AM086701)	120	62	65	61	63	67	69	54	67	71	79	76	56	71	88	86															
18	X._diffusum_(Brazil_AM086699)	122	63	64	58	62	64	65	50	67	70	79	74	55	71	83	81	11														
19	X._asta regiense_(Spain_KP268977)	132	113	108	113	102	116	103	105	116	105	106	112	106	119	122	122	116	116													
20	X._simile_(Slova kia_AM086708)	135	94	105	106	84	108	97	102	98	94	108	99	101	97	104	103	101	106	123												
21	X._peruvianum_ (Brazil_AM086712)	245	211	212	243	203	247	250	218	230	259	252	233	250	250	250	248	243	245	241	247											
22	X._peruvianum_(USA_ Georgia_AM086692)	272	234	241	255	224	265	268	242	254	279	267	258	264	266	269	269	263	266	256	264	72										
23	X._america num_(South_Africa_M K956813)	98	48	52	47	57	58	59	58	56	67	74	50	64	32	58	61	58	58	97	94	236	240									
24	X._america num_(South_Africa_AM086690)	119	57	59	53	60	64	67	63	62	75	83	56	71	34	67	69	66	63	114	98	248	268	4								
25	X. _americanum_(USA_Florida _AM086691)	273	243	244	260	234	284	279	247	266	286	282	256	274	274	272	272	279	277	267	278	260	291	239	276							
26	X._america num_ (USA_California _KX263065)	226	238	241	216	229	235	228	239	244	243	239	236	232	228	226	226	233	232	236	233	224	246	205	232	50						
27	X._america num_ (USA_California _KX26305 7)	232	247	249	221	235	247	239	245	250	253	248	242	238	236	236	236	243	242	245	239	224	249	213	240	6	47					
28	X._america num_(USA_Alabama_K X263058)	244	242	247	226	230	244	242	245	244	259	246	248	243	243	246	244	244	246	245	237	65	33	223	247	271	262	263				
29	X._america num_ (USA_California _KX263064)	247	237	243	227	225	245	241	243	245	259	250	249	246	244	247	245	247	249	251	236	63	28	223	248	271	257	256	35			
30	X._america num_ (USA_California _KX263060)	250	242	247	232	230	250	248	251	250	265	252	254	249	249	252	250	250	252	250	243	65	34	229	253	274	262	263	0	36		
31	X._america num_ (USA_California _KX263063)	253	242	247	235	230	253	251	251	253	268	255	257	252	252	255	253	253	255	252	246	65	35	232	256	277	262	263	0	37	0	
32	X._ina equale_ (Czech_Republic_H M163207)	264	249	258	258	233	279	270	259	286	287	278	272	272	265	267	267	269	269	283	270	259	290	241	272	76	69	67	279	281	282	285

Phylogenetic analysis of the aligned sequences revealed five major subclades within the studied *americanum*-group. They include: *X. americanum*, *X. californicum*, *Xiphinema* sp., *X. brevicolle* complex, and *X. pachtaicum*. *Xiphinema oxycaudatum* was closely related to *Xiphinema* sp. (Iran) and the Brazilian population of *X. peruvianum*. Within the 50% majority rule consensus MP tree, no significant difference was obtained in the two closely related species. However, sequences obtained from the *coxI* mitochondrial gene clearly discriminates *X. oxycaudatum* from other species within the *X. americanum*-group. The genetic relationship of this sequence with reference sequences obtained from the NCBI is illustrated in Figure 4.

## Discussion

Precise identification of nematode species and knowledge of their distribution is important for effective phytosanitary and management options. Species identification of nematodes within the *Xiphinema americanum* group is often difficult and complicated due to overlapping of morphological features and phenotypic plasticity. The taxonomy of this group of nematodes is often regarded as controversial and subjective (Luc et al. 1998; Orlando et al. 2016), and there is a possibility to confuse and misidentify species within the group.

Some key morphological features that have been frequently used as diagnostic keys for differentiating between species within the *Xiphinema americanum* group include the lip region, odontostyle length, position of C, tail shape, and length (Lamberti and Bleve-Zacheo 1979; Lamberti and Carone 1991). However, in more recent times, identification has been done in combination with molecular tools with indications of mitochondrial marker cytochrome oxidase subunit 1 (*coxI*) as a barcode for species identification and a tool for resolving the complexity in identifying cryptic *americanum* species (Palomares-Rius et al. 2017).

Although the molecular analysis, based on the D2D3 region of the nematodes species in the present study revealed low interspecific variation in the nematodes within the *X. americanum* group, two distinct clades were evident from the phylogenetic tree. *X. oxycaudatum* was separated in a group from other *Xiphinema* species with a strong statistical support. This was also evident from previous studies where low interspecific variation within the *X. americanum*-group has been reported (He et al. 2005; Orlando et al. 2016). They indicated that *X. americanum*-group species formed two highly supported clades, *X. americanum* and *X. pachtaicum* (sensu Lamberti and Ciano 1993). Oliveira et al. (2004) also obtained nearly identical result with analysis of the 18 rDNA sequences where species belonging to the *X. americanum*-group formed a single group separated from the other *Xiphinema* species. He however suggested that 18S rDNA does not provide a useful marker to discriminate *Xiphinema* in the *americanum* group at the species level. This was also confirmed by Zasada et al. (2014), who showed that 18S rDNA sequence data did not provide taxonomic clarity among some populations of *X. americanum*. In the present study, the sequences obtained from the ITS region were of poor quality and were not used for phylogenetic analysis.

The protein coding mitochondrial gene, cytochrome oxidase subunit I (*coxI*), has been described as a reliable and preferred molecular barcode and a useful tool for highlighting the intra-specie variation within some species of *X. americanum*-group (Lazarova et al. 2006; Gutiérrez-Gutiérrez et al. 2012; Lazarova et al. 2016; Orlando et al. 2016; Palomares-Rius et al. 2017). In the present study, *coxI* gene was used to reconstruct the phylogenetic relationship within the species; thus, in combination with morphological identification, it provided a useful tool for delimitation and discrimination of *X. oxycaudatum* from other species within the *americanum*-group.

This study represents the first report of *X. oxycaudatum* in association with honeybush in South Africa. The South African population is both morphometrically and genetically similar to *X. peruvianum*. Meza et al. (2011) indicated that a high homology exists between Chile population of *X. peruvianum* and *X. oxycaudatum* identified from Taiwan. The South African population are similar to *X. peruvianum* but are distinguished by their shorter odontostyle and tail shape. This nematode species has been reported in association with a wide range of cultivated plants from Nigeria, Kenya, Iran, Pakistan, Brazil, and Taiwan (Lamberti and Bleve-Zacheo 1979; Bos and Loof 1983; Coomans and Heyns 1997; Fadaei et al. 2003; Oliveira et al. 2003; Chen et al. 2005). Our record of *X. oxycaudatum*, in association with *Cyclopia* spp. from South Africa, will add a new record to this list.

Nematodes belonging to the *Xiphinema americanum*-group are cosmopolitan in their distribution and have phytopathological importance with some species being implicated as vectors of important plant viruses. High numbers of *X. oxycaudatum* that were recorded from the honeybush farmland in South Africa could have resulted from high multiplication rate of nematodes due to availability of a suitable host, presence of some attractants in the soil, and some edaphic factors. The occurrence of *X. oxycaudatum* in such high density recorded in this study is disturbing and suggests that a damage potential may exist, which could have future implications on the budding honeybush tea industry.

To our knowledge, this will be the first documented report of the occurrence of *X. oxycaudatum* in South Africa.
